# Biochemical reconstitution and genetic characterization of the major oxidative damage base excision DNA repair pathway in *Thermococcus kodakarensis*

**DOI:** 10.1016/j.dnarep.2019.102767

**Published:** 2019-12-05

**Authors:** Alexandra M. Gehring, Kelly M. Zatopek, Brett W. Burkhart, Vladimir Potapov, Thomas J. Santangelo, Andrew F. Gardner

**Affiliations:** a New England Biolabs, Inc., Ipswich, MA 01938, United States; b Department of Biochemistry and Molecular Biology, Colorado State University, Fort Collins, CO 80523, United States

**Keywords:** Archaea, DNA repair, Base excision repair (BER), 8oxo-guanine (8-oxoG), Nucleic acid enzymology, Genetics, DNA polymerase, DNA glycosylase, AP endonuclease

## Abstract

Reactive oxygen species drive the oxidation of guanine to 8-oxoguanine (8oxoG), which threatens genome integrity. The repair of 8oxoG is carried out by base excision repair enzymes in Bacteria and Eukarya, however, little is known about archaeal 8oxoG repair. This study identifies a member of the Ogg-subfamily archaeal GO glycosylase (AGOG) in *Thermococcus kodakarensis*, an anaerobic, hyperthermophilic archaeon, and delineates its mechanism, kinetics, and substrate specificity. TkoAGOG is the major 8oxoG glycosylase in *T. kodakarensis*, but is non-essential. In addition to TkoAGOG, the major apurinic/apyrimidinic (AP) endonuclease (TkoEndoIV) required for archaeal base excision repair and cell viability was identified and characterized. Enzymes required for the archaeal oxidative damage base excision repair pathway were identified and the complete pathway was reconstituted. This study illustrates the conservation of oxidative damage repair across all Domains of life.

## 1. Introduction

All cells must cope with reactive oxygen species generated both endogenously as a byproduct of cellular metabolism and exogenously from the environment. Reactive oxygen species cause the oxidation of DNA bases leading to the formation of a wide variety of DNA lesions, including 7,8-dihydro-8-oxoguanine (8oxoG), a product of guanine oxidation and one of the most abundant DNA lesions present in the cell [1,2]. In addition to base pairing with its Watson-Crick base cytosine, 8oxoG base pairs with adenine via a Hoogsteen interaction. Mispairing of adenine with 8oxoG by a DNA polymerase during DNA replication results in G:C to T:A transversions. Therefore, persistence of 8oxoG leads to increased mutational frequency and genome instability [1,3–6].

To repair 8oxoG, organisms depend on the base excision repair (BER) pathway. BER is initiated by a DNA glycosylase recognizing and removing the damaged base creating an apurinic/apyrimidinic (AP) site (reviewed in [7]). DNA glycosylases can be bifunctional with both glycosylase and AP lyase activities to cleave the phosphodiester backbone leaving a 5’-phosphate and either a 3’-α,β-unsaturated aldehyde (3’-UA) via β-elimination, or 3’-phosphate, via β,δ-elimination [7,8]. After recognition and removal of the damaged base by the glycosylase/lyase, an AP endonuclease converts the 3’-UA or 3’-phosphate to an extendable 3’-hydroxyl (3’-OH). A DNA polymerase synthesizes from the 3’-OH displacing the downstream strand, which is subsequently cleaved by a flap endonuclease or by a DNA polymerase 5’-3’ exonuclease, and then sealed by a DNA ligase [7,9].

The bacterial and eukaryotic oxidative damage BER pathways have been extensively studied and are initiated via a bifunctional DNA glycosylase, either a formamidopyrimidine DNA glycosylase (Fpg) or a member of the 8-oxoguanine DNA glycosylase (Ogg) family, respectively. Bacteria typically rely on Fpg for initiating repair of 8oxoG, while Archaea and Eukarya often rely on members of the Ogg family [10–12]. All Ogg enzymes belong to the helix-hairpin-helix (HhH) superfamily of DNA repair glycosylases [13,14]. Three distinct subfamilies of Ogg enzymes, Ogg1, Ogg2, and archaeal GO glycosylase (AGOG) share little sequence similarity [10]. Ogg1 is well-studied and is most commonly encoded in eukaryotes, including humans, and some bacterial species [13,15–22]. Ogg2 is most commonly found in Archaea but is also sparsely found in eukaryotic and bacterial genomes [23–29]. The distribution of AGOG, in contrast, is limited exclusively to archaeal species and AGOG is the least studied subfamily of Ogg enzymes [14].

Despite widespread distribution of AGOG in diverse archaeal lineages, only two representatives, from *Pyrobaculum aerophilum* (PaeAGOG) and *Thermococcus gammatolerans* (TgaAGOG), have been characterized *in vitro* [14,30–33], and very limited data regarding the *in vivo* importance of AGOG has been presented. The individual steps of archaeal BER of 8oxoG have been proposed, yet a complete reconstitution of any archaeal BER pathway – from recognition by the DNA glycosylase through nick sealing by a DNA ligase – has not been demonstrated.

In this study, we examined 8oxoG damage repair in *Thermococcus kodakarensis*, a hyperthermophilic, marine archaeon with a growth temperature range from 60 °C to 100 °C, and optimal growth at 85 °C [34]. The rate of spontaneous oxidation and formation of reactive oxygen species increases at higher temperatures [35,36], thus *T. kodakarensis* and other hyperthermophiles are predicted to encode robust oxidative DNA damage repair pathways to ensure genome stability [37,38]. 8oxoG lesions in the template strand stall both RNA and DNA polymerases, arguing that 8oxoG must be efficiently removed to limit mutagenic effects [6,38]. BER is the dominant repair pathway of 8oxoG in the *Thermococcales* [6,30] and here we characterize the complete pathway of oxidative damage repair in *T. kodakarensis* from 8oxoG recognition by a DNA glycosylase to nick sealing by a DNA ligase. We detail the *in vivo* roles of AGOG in *T. kodakarensis*, characterize AGOG enzymatic properties, identify the essential AP endonuclease (TkoEndoIV) as a critical constituent of archaeal BER, and reconstitute the complete 8oxoG BER pathway in Archaea.

## 2. Material and methods

### 2.1. Preparation of DNA substrates

The oligonucleotides used in this study were ordered from Integrated DNA Technologies (Coralville, IA) with 5’-FAM and 3’-ROX fluorophores for detection in capillary electrophoresis. The lesion containing substrates were prepared by annealing 1 μM of the labeled 60-nt lesion containing oligo (5’-FAM-TGGAGATTTTGATCACGGTAA-CCXATCAGAATGACAACAAGCCCGAATTCACCCAGGAGG-ROX-3’) where X is either 8oxoG, 8-oxoadenine, deoxyuridine (dU), deoxyinosine (dI), or deoxyxanthosine (dX) to 1.25 μM of the 60-nt complementary oligo (5’-CCTCCTGGGTGAATTCGGGCTTGTTGTCATTCTGATNGGTTACCGTGATCAAAATCTCCA-3’) where N is a canonical base in 1x annealing buffer (10 mM Tris-HCl pH 7.5 and 100 mM NaCl) at 85 °C for 5 min and allowing the reactions to slowly cool to room temperature.

### 2.2. T. kodakarensis growth and strain construction

*T. kodakarensis* strains were grown in artificial seawater supplemented with 5 g/L yeast extract, 5 g/L tryptone, and 2 g/L of sulfur at 85 °C [34]. Growth was measured by increases in optical density at 600 nm (OD_600_). The growth curve was performed with three independent biological replicates in triplicate.

Standard procedures were used to delete or attempt to delete TkoAGOG (encoded by TK0940) and TkoEndoIV (encoded by TK0170), respectively, from the genome of *T. kodakarensis* strain TS559 [39,40]. Briefly, a non-replicating plasmid was transformed into TS559 and was integrated into the genome at the loci of interest (TK0940 or TK0170) via recombination; transformants were identified by co-integration of a selectable marker restoring agmatine prototrophy. The intermediate strains containing the integrated plasmid were confirmed using diagnostic PCR on purified genomic DNA. The intermediate cells were then grown in the presence of agmatine as well as 6-methylpurine (the counter-selectable marker), and the plasmid was excised from the genomic loci. Again, diagnostic PCR using locus-specific primers on purified genomic DNA from these final strains was used to determine if the target locus was deleted from the genome or if the parent genome (TS559) had been restored. Whole genome sequencing using the PacBio RS II Sequencing platform (Pacific Biosciences, Menlo Park, CA) further confirmed the deletion of TK0940 (Fig. S1). TkoEndoIV was determined to be essential after > 30 final strains from two distinct intermediate strains resulted in restoration of the parent genome (TS559) [39].

### 2.3. Analysis of mutational spectra

The wild-type and deletion *T. kodakarensis* genomic libraries were sequenced using PacBio RSII instrument. Following sequencing, the bioinformatics analysis of mutational events was done as described previously with modifications [41]. In brief, the high-accuracy consensus sequences were built using PacBio tools and mapped to a reference *T. kodakarensis* genome (GenBank NC_006624) using BLASR aligner. A number of filtering steps were used to avoid sequencing artifacts. At least 15 passes were required to build consensus sequences and only high-quality bases were used in the analysis (Phred quality score of 93). The consensus reads were required to map unambiguously with the mapping quality score of 254 while all supplementary (chimeric) alignments were discarded. Additionally, clipped aligned consensus reads were discarded to avoid chimeric reads arising during PacBio library preparation.

### 2.4. Cloning, expression, and purification of archaeal proteins

An *Escherichia coli* codon optimized version of the genes encoding TkoAGOG (GenBank BAD85129.1), and TkoEndoIV (GenBank BAD84359.1) from *T. kodakarensis* were synthetically constructed and cloned into pAII17 vector via the Ndel and BamHI sites (Genscript, Piscataway, NJ). The plasmid containing TkoAGOG was transformed into BL21(DE3) *E. coli* cells while the plasmid containing TkoEndoIV was transformed into T7 Express lysY/I^q^ competent cells (New England Biolabs, Ipswich, MA).

The cells were grown at 37 °C in LB media supplemented with 0.1 mg/mL of ampicillin until an OD_60_o of 0.6 was reached. Protein expression was induced with 0.4 mM IPTG and cultures were allowed to continue growing at 37 °C for 3 h. The cells were harvested by centrifugation at 4500 × *g* for 20 min. The cell pellet was suspended in Buffer A (20 mM Tris-HCl pH 7.5 and 50 mM NaCl), lysed using a constant cell disruptor (Constant Systems LTD, Northants, UK), and heat treated at 80 °C for 20 min. The cell debris was removed via centrifugation at 35,000 × *g* for 15 min.

For TkoAGOG, the clarified cell lysate was passed through a DEAE column and the flow-through was collected. The flow-through was loaded onto a HiPrep Heparin FF 16/10 column (GE Life Sciences, Pittsburgh, PA) and eluted in fractions with a buffer gradient from 50 mM to 1 M NaCl. TkoAGOG containing fractions were identified by SDS-PAGE, pooled and dialyzed to 100 mM KC1, 10 mM Tris-HCl, 1 mM DTT, 0.1 mM EDTA, and 50 % glycerol at pH 7.4.

For TkoEndoIV, the clarified cell lysate was passed through a DEAE column and the flow-through was collected. The flow-through was loaded onto a HiPrep Heparin FF 16/10 column (GE Life Sciences, Pittsburgh, PA) and eluted in fractions with a buffer gradient from 50 mM to 1 M NaCl. Fractions containing TkoEndoIV were identified by SDS-PAGE, pooled and diluted to 50 mM NaCl and were again loaded onto a HiPrep Heparin FF 16/10 column. A final elution was done using a buffer gradient from 50 mM to 1 M NaCl. TkoEndoIV containing fractions were identified by SDS-PAGE, pooled and dialyzed to 100 mM KC1, 10 mM Tris-HCl, 1 mM DTT, 0.1 mM EDTA, and 50 % glycerol at pH 7.4.

Other replication proteins, PCNA, RFC, DNA Ligase, Fen1, Pol B from *T. kodakarensis* and Pol D from *Thermococcus* sp. 9°N were purified as previously described [42–45].

### 2.5. Glycosylase and AP lyase activity of AGOG

Bifunctional DNA glycosylases have base removal glycosylase activity and AP lyase activity that leading to a break in the DNA backbone leaving a 5’-phosphate and either a 3’-UA, via α-elimination, or a 5’-phosphate via β,δ-elimination (Fig. 2A). To confirm the presence of glycosylase and AP lyase activity in AGOG, a 20 μ L reaction containing 20 nM 8oxoG:C dsDNA and 100 nM AGOG in 1x Thermopol Buffer was incubated at 65 °C for 30 min. For a β,δ-elimination positive control, the above reaction was performed with Fpg instead of AGOG. For a β-elimination positive control, 20 nM dU:G dsDNA and 100 nM of Uracil DNA glycosylase (New England Biolabs, Ipswich, MA) and Endonuclease III (New England Biolabs, Ipswich, MA) in IX Thermopol Buffer were incubated at 37 °C for 30 min [8,46]. All reactions were quenched by the addition of equal volume of 85 % formamide and 50 mM EDTA, followed by dilution in water to bring the final concentration of DNA to 2 nM. A 3730x1 Genetic Analyzer (Applied Biosystems) was used for capillary electrophoresis and the resultant fluorescent peaks were analyzed using Peak Scanner software version 1.0 (Applied Biosystems) [42].

### 2.6. Trapping of AGOG active site Schiff-base intermediate

Sodium borohydride trapping of the Schiff-base intermediate is a well-established technique to identify the amino acid residue responsible for formation of the Schiff-base intermediate, a covalent linkage between the DNA and enzyme [8,47]. Prior to the trapping of the Schiff-base intermediate, TkoAGOG was dialyzed into sodium acetate containing storage buffer (10 mM sodium acetate pH 5.5, 100 mM KC1, 1 mM DTT, 0.1 mM EDTA, 0.1 % Triton X-100, and 50 % glycerol). A 60-bp 8oxoG:C dsDNA was prepared as described above in 10 mM sodium acetate pH 5.5 and 20 mM NaCl.

Immediately prior to trapping of the Schiff-base intermediate, a fresh solution of 200 mM NaBH_3_CN in sodium acetate buffer (10 mM sodium acetate pH 5.5 and 20 mM NaCl) was prepared. Equimolar DNA and enzyme (5 μM final concentration) were mixed in sodium acetate buffer and NaBH_3_CN (final concentration 33 mM) was added. The reaction was then heated to 65 °C for 1 h. After incubation the products were analyzed using a 4–20 % SDS polyacrylamide gel.

### 2.7. Multiple turnover kinetics of TkoAGOG

To assess the turnover of TkoAGOG and determine if product release was the rate-limiting step of the reaction, a multiple-turnover kinetic assay was performed where the DNA substrate was in excess of TkoAGOG. A 200 μL reaction was made in lx Thermopol buffer containing 100 nM of 60-bp 8oxoG:C dsDNA and 5 nM TkoAGOG (20-fold excess substrate) and heated to 65 °C. 10 μL aliquots were removed and stopped at the appropriate time points (15 s – 10 min) with equal volume 0.1 N NaOH, 0.25 % SDS and then neutralized with equal volume 1 M Tris-HCl pH 7.5. The reactions were desalted using Optima DTR 96-well plates (EdgeBio, San Jose, CA) and analyzed using capillary electrophoresis as described above.

The concentration of product was determined and graphed as a function of time and fit to a linear equation to obtain the AGOG steady-state rate (k_ss_) using Kaleidagraph (Synergy Software, Reading, PA). The data were fit to a linear equation due to the lack of a pre-steady-state burst. All kinetic assays were performed at least 3 times to ensure reproducibility.

### 2.8. Single turnover kinetics of AGOG

To determine the rate of TkoAGOG glycosylase base removal activity, single-turnover kinetic assays were performed with TkoAGOG in excess of the substrate. A control experiment with a 5-fold molar excess of TkoAGOG demonstrated that the substrate was saturated. A 200 μL reaction in 1x Thermopol buffer containing 20 nM of 60-bp 8oxoG:N dsDNA was then heated to 65 °C, and 100 nM TkoAGOG (final concentration) was added to start the reaction. 10 μL aliquots were removed, quenched, cleaned-up, and analyzed as described above.

The concentration of product was graphed as a function of time and fit to a single-exponential equation (Eq. (1)) to obtain the AGOG pre-steady-state rate (k_pss_) using Kaleidagraph (Synergy Software, Reading, PA). All kinetic assays were performed at least 3 times to ensure reproducibility.

(1) $$\left[\text{Product}]=A(1-\exp(k_{\mathrm{obs}}\times t)\right)$$

### 2.9. Assessing 8-oxo-guanine activity in T. kodakarensis cell extract

Cell extracts from the parent strain, TS559, and ΔAGOG were prepared by resuspending 1 g of cell paste in 1 mL of Buffer A (20 mM Tris-HCl pH 7.5, 50 mM NaCl). The cells were then sonicated for 2 min and centrifuged for 10 min at 14,000 x g to remove cell debris. Glycerol was added to the cell extract to a final concentration of 50 %. The amount of enzyme activity in cell extracts were normalized by detecting RNaseH2 activity as previously described [41]. The 60-bp 8oxoG:C dsDNA substrate (40 nM) was incubated with *T. kodakarensis* TS559 or ΔAGOG cell extracts in 1x ThermoPol buffer (20 mM Tris-HCl, 10 mM (NH_4_)_2_SO_4_, 10 mM KC1, 2mM MgSO_4_, pH 8.8 at 25 °C) at 65 °C for 30 min. As a control, 100 nM of purified recombinant TkoAGOG was added to the cell extract of ΔAGOG. The reactions were quenched with 0.1 N NaOH and 0.25 % SDS and then neutralized with 1 M Tris-HCl pH 7.5. The reactions were desalted using Optima DTR 96-well plates (EdgeBio, San Jose, CA) and analyzed using capillary electrophoresis.

### 2.10. Characterization of TkoEndoIV

The activity of TkoEndoIV was characterized as follows. To generate an AP-site containing dsDNA, a 60-bp deoxyuridine:G dsDNA was pretreated with UDG for 30 min at 37 °C in 1x Thermopol buffer to produce an AP site. A 100 μL reaction was made containing 1x Thermopol buffer, 100 nM of substrate DNA, and 20 nM TkoEndoIV. The total reaction was heated to 65 °C, and 10 μL aliquots were taken at the appropriate time points (15 s – 30 min). The reactions were stopped with equal volume 50 mM EDTA and analyzed using capillary electrophoresis as described above.

### 2.11. Reconstitution of the 8-oxo-guanine BER pathway

8oxoG BER pathway reconstitution reactions were performed by pre-incubating 2.5 nM 60-bp 8oxoG:C dsDNA with 10 nM each of TkoAGOG and TkoEndoIV in 1x Thermopol buffer at 65 °C for 30 min. Following pre-incubation, PCNA-1 (20 nM), RFC (10 nM), Pol B exo- (2.5 nM), Pol D exo- (2.5 nM), Fen1 (25 nM), and DNA ligase (25 nM), ATP (2 mM), dNTPs (0.1 mM), and MgSO_4_ (8mM) were added and incubated for 5 — 15 min. All proteins are from *T. kodakarensis* except Pol D exo- which is from *Thermococcus* sp. 9°N [42,43,48]. The reaction was then incubated at 65 °C, stopped with equal volume 50 mM EDTA, and analyzed using capillary electrophoresis as previously described.

## 3. Results

### 3.1. Identification and biochemical characterization of AGOG from T. kodakarensis

**Fig. 1.**
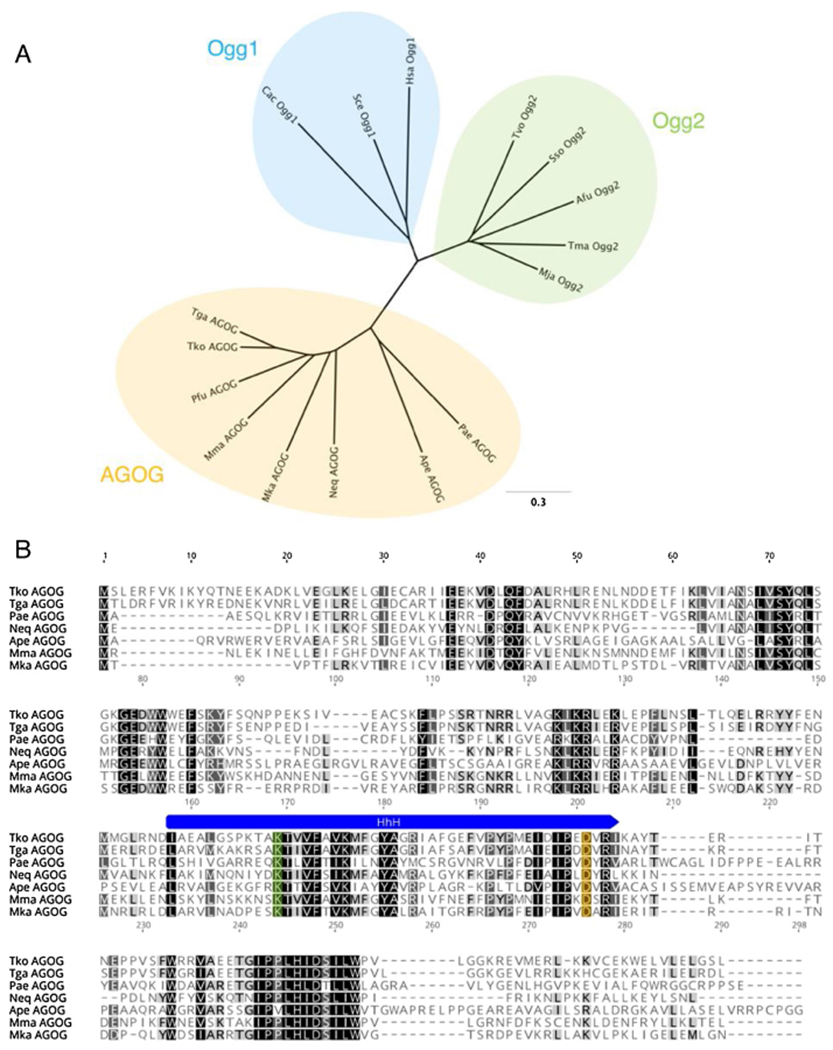
*T. kodakarensis* AGOG is a member of the AGOG subfamily of Ogg enzymes. A, Unrooted phylogenetic tree of Ogg family enzymes with the 3 distinct subfamilies highlighted: Ogg1 (blue), Ogg2 (green), and AGOG (yellow). The tree was generated using Geneious v11.0.2. B, Amino acid sequence alignment of members of the AGOG subfamily. The conserved Helix-hairpin-Helix (HhH) is marked in blue, the catalytic lysine residue in orange, and the active site aspartic acid in green. The species abbreviations and GenBank ascension numbers used are as follows: Archaea: Afu, *Archaeoglobus fulgidus* (Ogg2, AAB90876); Ape, *Aeropyrum pernix* (AGOG, BAA79686); Mja, *Methanocaldococcus jannaschii* (Ogg2, AAB98720); Mma, *Methanococcus maripaludis* (AGOG, CAF29860); Neq, *Nanoarchaeum equitans* (AGOG, AAR39356); Mka, *Methanopyrus kandleri* (AGOG, AAM01756); Pae, *Pyrobaculum aerophilum* (AGOG, AAL64050); Pfu, *Pyrococcus furiosus* (AGOG, AAL81028); Sso, *Saccharolobus solfataricus* (Ogg2, AAK41186); Tga. *Thermococcus gammatolerans* (AGOG, ACS34155); Tko, *Thermococcus kodakarensis* (AGOG, BAD85129); Tvo, *Thermoplasm volcanium* (Ogg2, WP_010916318); Bacteria: Cac, *Clostridium acetobutylicym* (Ogg1, NP_349313); Tma, *Thermotoga maritima* (Ogg2, AGL50755); Eukarya: Hsa, *Homo sapiens* (Ogg1, NP_002533); Sce, *Saccharomyces cerevisiae* (Ogg1, NP_013651).

The three distinct subfamilies of Ogg enzymes (Ogg1, Ogg2, and AGOG) share limited sequence similarity [10]. To identify an 8oxoG-specific DNA glycosylase in *T. kodakarensis*, we searched sequence databases for putative homologs of known archaeal Ogg2 and AGOG enzymes, as well as eukaryotic Ogg1. The *T. kodakarensis* genome lacks obvious Ogg1 or Ogg2 homologs, but gene TK0940 encodes a putative AGOG. TK0940p (hereafter termed TkoAGOG) is similar to AGOG enzymes from other *Thermococcales* and is 30 % identical to the previously described AGOG from *P. aerophilum* [14] (Fig. 1). The overall sequence homology between members of the AGOG subfamily is often low, as is the case for the AGOG homologs from *T. kodakarensis* and *P. aerophilum*, however the HhH motif and the active site lysine and aspartic acid are sufficiently conserved to identify AGOG homologs [14,31,32,37] (Fig. 1).

**Fig. 2.**
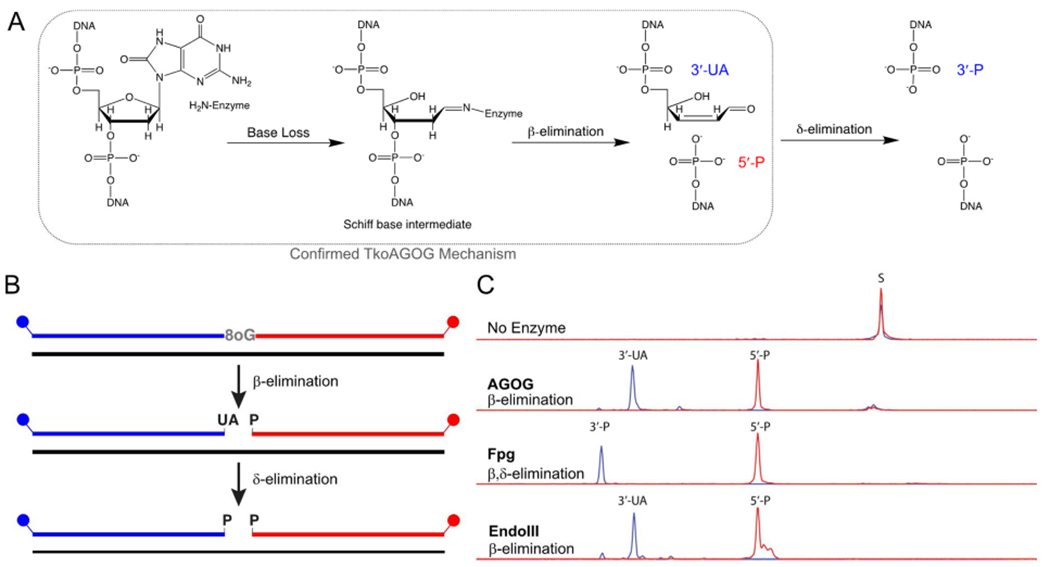
TkoAGOG is a bifunctional 8oxoG DNA glycosylase. *A*, Proposed enzymatic mechanism of a bifunctional 8oxoG DNA glycosylase, where 8oxoG nucleobase loss is followed by a Schiff base intermediate that undergoes β-elimination and for some DNA glycosylases such as Fpg further δ-elimination. The confirmed TkoAGOG mechanism is boxed in grey *B*, A 60-nt, 5’-FAM (blue), 3’-ROX (red) labeled dsDNA substrate with a centralized 8oxoG:C (or dU:G) was incubated for 30 min with either TkoAGOG at 65 °C, Fpg at 37 °C, or UDG/EndoIII at 37 °C and quenched with Formamide + EDTA, allowing for visualization of the base excision glycosylase and AP lyase activities. *C*, The conversion of 60-nt substrate to the 5’-FAM and 3’-ROX products after incubation with TkoAGOG, Fpg, or UDG/EndoIII.

To confirm the activity of TK0940 as an 8oxoG DNA glycosylase, we recombinantly expressed and purified TkoAGOG for detailed characterization. Incubation of TkoAGOG with the dual-labeled 8oxoG:C dsDNA substrate led to cleavage of the lesion containing strand, confirming TkoAGOG both removes the 8oxoG base and breaks the DNA backbone via DNA glycosylase and AP lyase activity, respectively (Fig. 2). To define the products of TkoAGOG AP lyase activity, two controls were performed. Endo III cleaves AP sites via β-elimination to produce a gap with a 3’-UA and a 5’-phosphate [46] while Fpg cleaves 8oxoG:C dsDNA via β,δ-elimination to produce a gap with both 3’-and 5’-phosphates [8,12] (Fig. 2). The products left by TkoAGOG are identical to those of Endo III, demonstrating TkoAGOG undergoes only β-elimination producing a gap with 3’-UA and a 5’-phosphate. Therefore, TkoAGOG and the previously described PaeAGOG and TgaAGOG are bifunctional glycosylases, capable of removing the 8oxoG damaged base via glycosylase activity and cleaving the DNA backbone via AP lyase β-elimination activity [14,30,32,33] (Fig. 2).

It is important to note that the 3’-UA β-elimination product can spontaneously (not enzymatically) undergo δ-elimination, and we routinely observe a small fraction, < 5 %, of TkoAGOG 3’-UA product converted to the 3’-phosphate. Because of this conversion, all quantitation of AGOG activity was completed using the 3’-ROX labeled strand.

Additionally, the proposed TkoAGOG mechanism goes through a Schiff-base intermediate involving a covalent attachment of the active site lysine to the DNA (Fig. 2A). Incubation of TkoAGOG with the dual-labeled 8oxoG:C dsDNA substrate in the presence of sodium cyano-borohydride reduced the Schiff-base intermediate. TkoAGOG remained covalently attached to the DNA, as visualized by denaturing PAGE, providing confirmation of the Schiff-base intermediate and further support of the enzymatic mechanism [47] (Fig. S2).

**Fig. 3.**
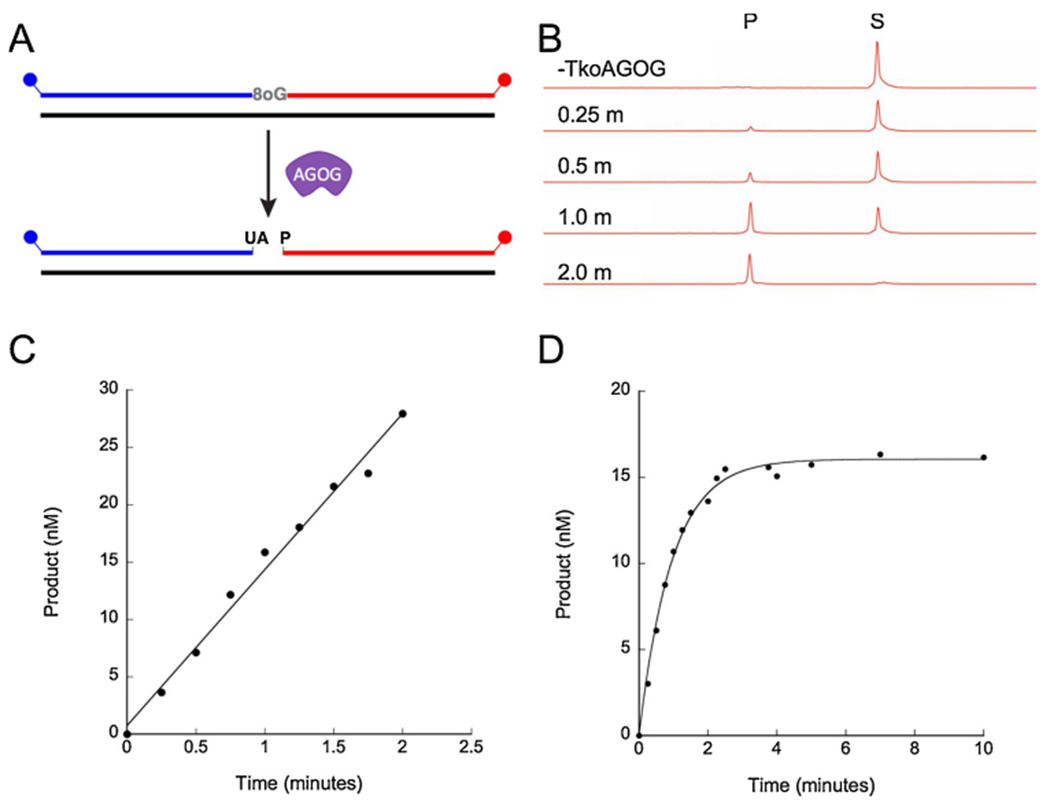
Steady-state and pre-steady-state kinetics of TkoAGOG. *A*, A 60-nt, 5’-FAM (blue), 3’-ROX (red) labeled dsDNA substrate with a centralized 8oxoG was incubated with TkoAGOG at 65 °C and quenched with NaOH + SDS allowing for visualization. *B*, The conversion of the 60-nt substrate to the 36-nt 3’-ROX product by TkoAGOG over time. *C*, A representative graph of a TkoAGOG steady-state multiple turnover experiment ([DNA] > > [TkoAGOG]). The amount of product formed was calculated using the 3’-ROX labeled fragment, and data was fit to a linear line. *D*, A representative graph of a TkoAGOG pre-steady-state single turnover experiment ([TkoAGOG] > > [DNA]). The product formed was calculated using 3’-ROX labeled fragment, and data was fit to Eq. (1).

A kinetic characterization of TkoAGOG was performed to understand substrate preference and rates. Varying ratios of TkoAGOG and the 60-nt 8oxoG:C dsDNA substrate were incubated at 65 °C and quenched at various time points with NaOH and SDS to convert all sites lacking an 8oxoG base to strand breaks (Fig. 3A,B). Characterization of all bacterial and eukaryotic 8oxoG DNA glycosylases by steady-state multiple turnover kinetics ([substrate] > > [enzyme]) suggests that product release is the rate limiting step due to the presence of a pre-steady-state burst of activity. Therefore, we first aimed to determine the rate limiting step of the TkoAGOG mechanism. However, under steady-state multiple turnover conditions, similar to those used to characterize eukaryotic and bacterial 8oxoG glycosylases, we did not observe a pre-steady-state burst, but rather a linear line, where the slope represents the steady-state rate (k_ss_) (0.76 ± 0.22 min^−1^) (Fig. 3C). The absence of a burst of product formation under steady-state conditions suggests that the rate of glycosylase base removal or a step prior, such as substrate binding, is rate limiting.

To measure the rates leading up to and including glycosylase base removal, we performed pre-steady-state single turnover kinetics ([enzyme] > > [substrate]). The pre-steady-state rate (k_pss_) of AGOG is 1.21 ± 0.18 min^−1^ on dsDNA containing 8oxoG:C (Fig. 3E) and is three-fold slower on ssDNA containing 8oxoG (0.41 ± 0.04min^−1^) (Table 1). Furthermore, the k_pss_ and k_ss_ are similar and within error of each other confirming the rate of glycosylase base removal or a step prior, such as substrate binding, is rate limiting.

8oxoG base-pairs with either cytosine, the canonical base, or with adenine via a Hoogsteen base-pair. Ogg1 family enzymes have a strong substrate preference for cleaving 8oxoG opposite cytosine compared to 8oxoG paired with adenine, guanosine or thymine [21,31,49,50]. In contrast, Ogg2 and AGOG family enzymes cleave 8oxoG:C and 8oxoG:A with similar rates (within two-fold). To determine if TkoAGOG discriminates between different 8oxoG base-pairs, substrates containing 8oxoG across from either adenine, cytosine, thymine, or guanine were tested under single turnover conditions. Although TkoAGOG removes 8oxoG across from all DNA lesions, a two-fold preference for 8oxoG base-paired with cytosine, thymine or guanine was observed compared to 8oxoG paired with adenine. The k_pss_ of TkoAGOG for all of the DNA substrates is similar to that of PaeAGOG (Table 1) [14].

We next determined the range of DNA lesions cleaved by TkoAGOG. Incubation of TkoAGOG with AP site containing dsDNA substrate resulted in the formation of the β-elimination product, suggesting the AP lyase activity of TkoAGOG can be uncoupled from the glycosylase activity (Fig. S3). TkoAGOG was further tested against a suite of DNA substrates containing a variety of DNA lesions including 8-oxo-adenine, dU, dI, or dX but no activity was detected even after extended incubation times (Fig. S3). Therefore, TkoAGOG, has a very narrow substrate specificity for only 8oxoG and AP site containing DNA. These results are consistent with previous experiments using PaeAGOG [32] and in contrast to bacterial Fpg which removes a wide variety of DNA lesions including 8-oxoadenine, fapy-guanine, methyl-fapy-guanine, fapy-adenine, aflatoxin B1-fapy-guanine, 5-hydroxy-cytosine and 5-hydroxy-uracil [51].

### 3.2. In vivo role of TkoAGOG

**Fig. 4.**
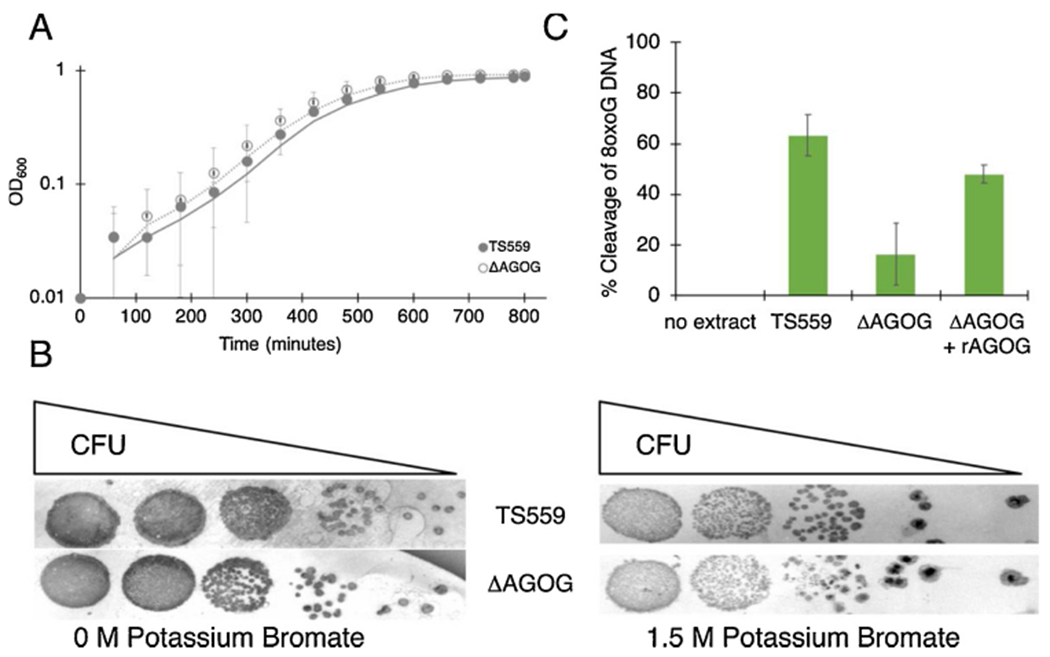
TkoAGOG is the major 8oxoG BER enzyme. *A*, Deletion of TkoAGOG (open circles, dashed line) does not affect cellular growth compared to the parent strain TS559 (closed circles, solid line). *B*, Deletion of TkoAGOG does not result in any phenotypic difference following exposure to potassium bromate. *C*, Cleavage of dsDNA containing 8oxoG by *T. kodakarensis* cellular extracts. As a positive control, recombinant TkoAGOG was added to the ΔAGOG extract.

The entire gene encoding TkoAGOG (gene TK0940) was marker-lessly deleted from the *T. kodakarensis* genome using established genetic techniques demonstrating TK0940 is a non-essential gene [39,40]. Further, under standard growth conditions, no apparent phenotypic consequence was observed when TkoAGOG was deleted (Fig. 4A). Potassium bromate specifically induces oxidative DNA damage while having minimal effect on other cellular processes [52,53]. There was no observable difference in cell susceptibility to potassium bromate between the parent strain, TS559, and ΔAGOG (Fig. 4B).

TkoAGOG is the only AGOG family member and sole annotated DNA glycosylase encoded in the *T. kodakarensis* genome specific for 8oxoG repair. To probe if other redundant enzymes recognize and repair 8oxoG (in addition to TkoAGOG), cellular extracts from both parental TS559 and ΔAGOG strains were assayed for activity on the dsDNA 8oxoG:C substrate. As expected, extracts prepared from parental TS559 cells (containing TkoAGOG) cleaved the majority 8oxoG:C DNA substrates while extracts derived from cells lacking TkoAGOG (ΔAGOG) had minimal cleavage activity (less than 10 %). These data confirm that TkoAGOG is a major 8oxoG repair enzyme in *T. kodakarensis* (Fig. 4C). The remaining residual 8oxoG cleavage activity in the ΔAGOG extract suggests *T. kodakarensis* encodes at least one other enzyme capable of acting on 8oxoG.

### 3.3. Mutational spectra of T. kodakarensis strains

In *E. coli* and *Saccharomyces cerevisiae* strains lacking oxidative repair enzymes, 8oxoG:A mismatches accumulate and result in G to T and C to A transversions [54–57]. To analyze the genome-wide mutational spectra in *T. kodakarensis* strains TS559 (parent) and ΔAGOG, both genomes were sequenced and the total number of nucleotide substitution events was calculated as compared to the reference. Of these substitution events (39 in ΔAGOG and 35 in TS559), 8 % in ΔAGOG and 20 % in TS559 were G to T or C to A transversions (Table 2). Furthermore, for all substitution events, the mutational spectra between TS559 and ΔAGOG strains were similar (Table 2). These results suggest that cells lacking TkoAGOG do not accumulate 8oxoG in the genome and likely have another mechanism that facilitates removal of 8oxoG.

### 3.4. Identification of the T. kodakarensis AP endonuclease

In Bacteria and Eukarya, an AP endonuclease is required to convert the resulting DNA glycosylase product to a 3’-OH, allowing extension by a DNA polymerase [58–61]. Using a homology-based search [62–67], we identified the major AP endonuclease in *T. kodakarensis* encoded by TK0170, herein named TkoEndoIV. TkoEndoIV shares all nine of the conserved metal-binding residues with other EndoIV homologs from both bacteria and archaea (Fig. S4). *T. kodakarensis* encodes an additional AP endonuclease homolog, encoded by TK1165, however this enzyme had no detectible activity on AP site containing DNA (data not shown). This is consistent with results from *Pyrococcus furiosus* [62].

**Fig. 5.**
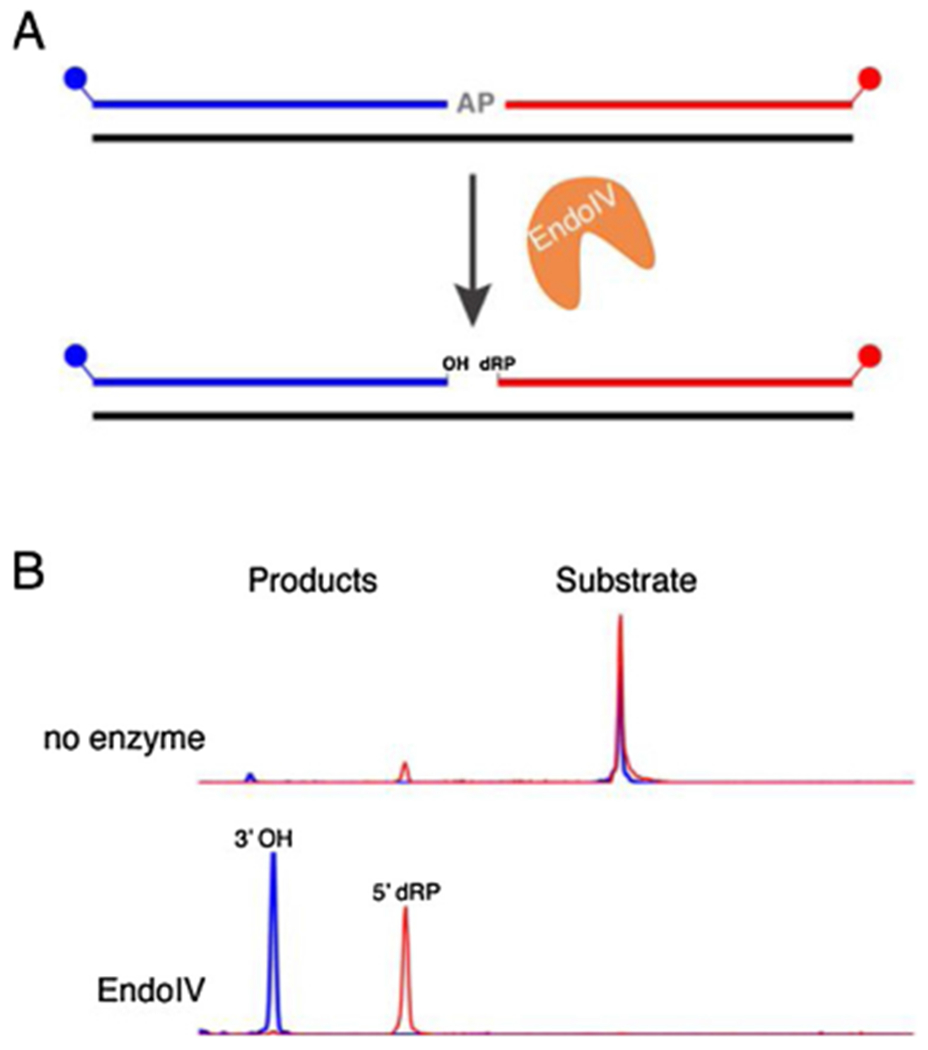
TkoEndoIV cleaves DNA at an AP site. *A*, A 60-nt, 5’-FAM (blue), 3’-ROX (red) labeled dsDNA substrate with a centralized AP site was incubated with TkoEndoIV at 65 °C and quenched with EDTA. *B*, Capillary electrophoresis traces showing the conversion of the 60-nt substrate to the 3’-ROX product after 10 min incubation with TkoEndoIV.

**Fig. 6.**
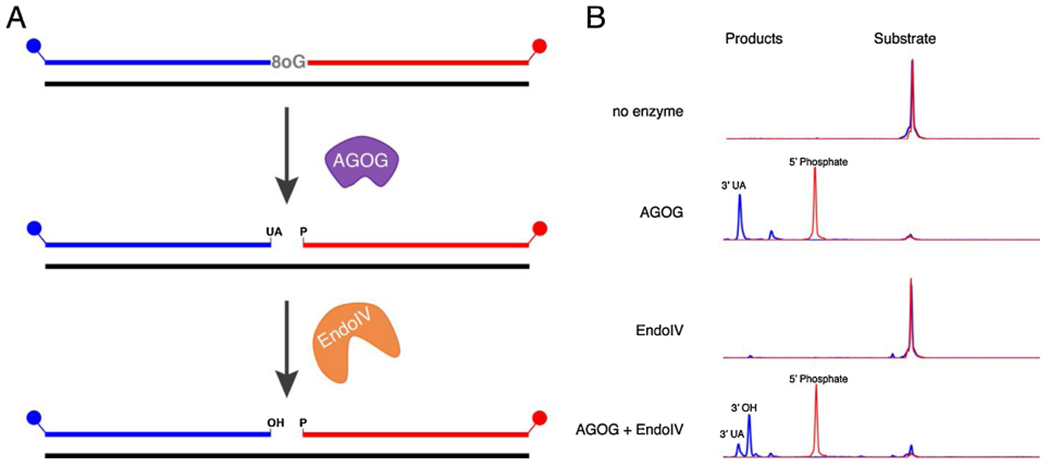
TkoAGOG and TkoEndoIV are both required to generate a 3’-OH from the site of an 8oxoG lesion. *A*, A 60-nt, 5’-FAM (blue), 3’-ROX (red) labeled dsDNA substrate with a centralized 8oxoG was incubated with either TkoAGOG, TkoEndoIV, or both TkoAGOG and TkoEndoIV at 65 °C and quenched with EDTA. *B*, Capillary electrophoresis traces highlighting the conversion of the 60-nt substrate to the 5’-FAM-labeled products, 3’-UA and 3’-OH after 30 min incubation with TkoAGOG and TkoEndoIV.

TkoEndoIV efficiently cleaves AP sites to produce a 3’-OH and 5’-deoxyribose-phosphate (5’-dRP) (Fig. 5). Importantly, we confirmed that TkoEndoIV also converts the 3’-UA left by TkoAGOG to a 3’-OH. (Fig. 6). Consistent with other archaeal EndoIV enzymes, TkoEndoIV showed no detectible activity on deamination substrates including dU, dl, or dX (Fig. S3) [62,66].

To probe the *in vivo* role of TkoEndoIV, genetic knockouts were attempted. Using established genetic techniques, the gene encoding TkoEndoIV could not be deleted from the genome and is essential for cell viability, confirming TkoEndoIV plays a crucial role *in vivo* [39,40]. TkoEndoIV likely plays a role in many other DNA repair pathways including deamination and depurination which depend on AP site cleavage [68].

Studies in Eukarya demonstrate the AP endonuclease (APE1), homologous in function to TkoEndoIV, increases the turnover rate of Ogg1 and may increase oxidative damage repair efficiency [25,69]. Unlike eukaryotic APE1, TkoEndoIV does not increase turnover of TkoAGOG (Fig. S5). Therefore, TkoAGOG and TkoEndoIV function independently, however both enzymes are required for oxidative damage BER.

### 3.5. In vitro reconstitution of the 8oxoG base excision repair pathway

**Fig. 7.**
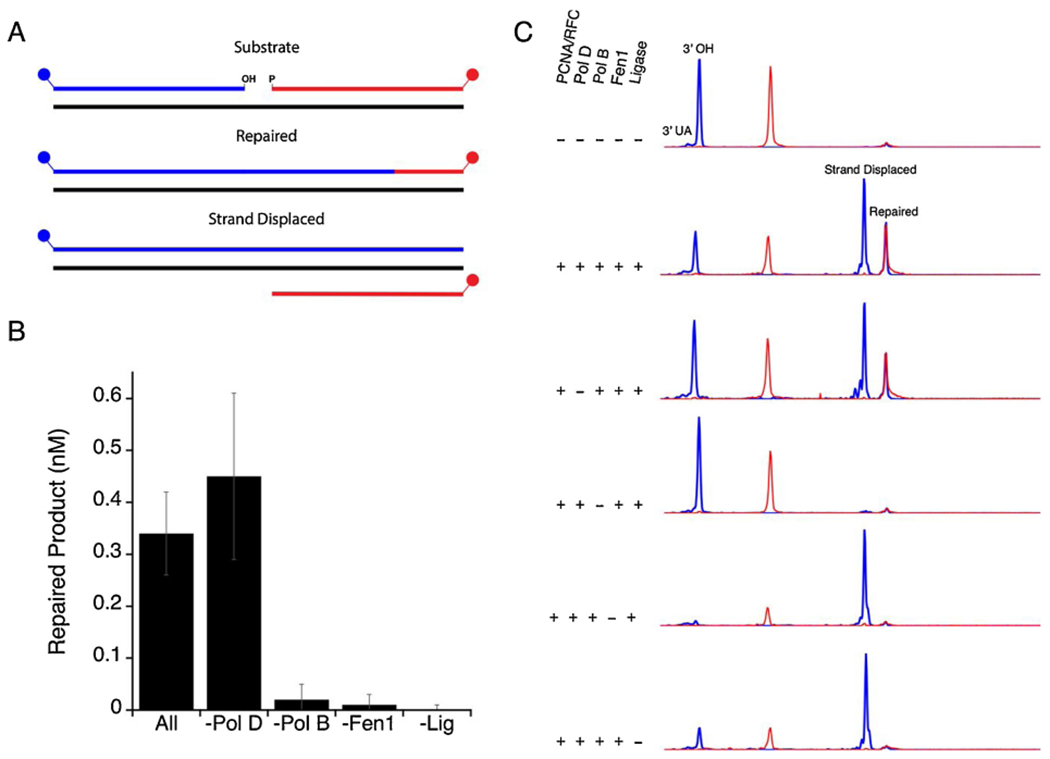
Archaeal 8oxoG BER reconstituted *in vitro. A*, Schematic of the reaction products observed. *B*, Quantification of the amount of repaired product after 15 min. *C*, DNA substrates were pre-incubated with TkoAGOG and TkoEndoIV leaving a 3’-OH or a 3’-UA with a FAM (blue) label. DNA replication proteins were then added as shown on the left and incubated at 65 °C for 15 min. The repair was monitored by the appearance of the 60-nt dual-labeled FAM/ROX product (repaired) and the 60-nt FAM only labeled product (strand displaced).

As shown by genetic and biochemical data, TkoAGOG is the major enzyme in *T. kodakarensis* required to initiate 8oxoG damage repair. Experiments were performed to determine the *Thermococcus* enzymes required for complete 8oxoG repair *in vitro.* The 8oxoG dsDNA substrate was pre-incubated with TkoAGOG and TkoEndoIV to remove 8oxoG and create a 1-nt gap with a 3’-OH and a 5’-phosphate termini (Fig. 7). When incubated with DNA polymerases B (Pol B) and D (Pol D), PCNA, RFC, Fen1 and DNA ligase, full repair was observed by the formation of a dual FAM and ROX labeled 60-mer product. In the absence of Pol D, the formation of repaired product was not changed, however in the absence of Pol B, repaired product was not observed. Because of strong Pol B strand displacement activity, a significant percentage of the 5’-FAM fragment is extended to the end of the 60-nt template before Fen1 cleavage or ligation can occur. Furthermore, in the absence of either Fen1 or DNA ligase, only the strand displaced product, not the repaired product was observed. The presence of PCNA and RFC had no effect on the formation of either the repaired or strand displaced product (Fig. S6). The results obtained demonstrate that for complete 8oxoG repair, TkoAGOG excises 8oxoG and cleaves the DNA backbone, TkoEndoIV converts the 3’-UA to a 3’-OH, strand displacement synthesis by Pol B creates a 5’ flap structure that is cleaved by Fen1 to create a nick that is sealed by DNA ligase.

## 4. Discussion

The extreme environment in which *T. kodakarensis* thrives requires that cells survive constant bombardment of DNA by innumerable DNA damaging agents including reactive oxygen species. 8oxoG is generated by reactive oxygen species and is one of the most common DNA lesions [1,2]. To cope with the presence of 8oxoG in genomic DNA, cells have efficient DNA repair pathways, including BER, to prevent DNA damage and genome instability.

Here we identified the major 8oxoG BER glycosylase, TkoAGOG, as well as the major AP endonuclease, TkoEndoIV, in *T.* kodakarensis. Our kinetic investigation shows the rate limiting step of 8oxoG removal by TkoAGOG is glycosylase base removal or a step prior. For all other kinetically characterized Ogg enzymes, product release is rate-limiting. In Eukarya, slow Ogg1 product release is stimulated by APE1 and may be crucial for coordination of Ogg with downstream BER enzymes [69,70]. In contrast, TkoAGOG is not stimulated by TkoEndoIV (Fig. S5) and the independent activities of TkoAGOG and TkoEndoIV suggest a unique mechanism for TkoAGOG initiated BER in *T. kodakarensis.* The possibility of coordination of TkoAGOG with other BER enzymes warrants further investigation.

The 3’-UA left by TkoAGOG β-elimination cannot be extended by a DNA polymerase thus must be converted to 3’-OH by TkoEndoIV. Likewise, additional enzymes in bacteria (ExoIII or EndoIV) and eukaryotes (APE1) convert the non-extendable 3’-termini to 3’-OH [12]. TkoEndoIV is crucial for TkoAGOG initiated BER, and may prove critical for all DNA glycosylase initiated BER in *T. kodakarensis* as cell viability is absolutely dependent on the presence of TkoEndoIV. The combined genetic and biochemical data suggest that TkoEndoIV, like other EndoIV homologs, may have critical roles not only in BER but likely in resolving a variety of 3’ modified termini resulting from DNA breaks or other repair pathways [58,59,64].

Both TkoAGOG and TkoEndoIV are necessary for BER of 8oxoG, but the *in vitro* reconstitution studies demonstrate complete repair is also dependent on a DNA polymerase, Fen1, and DNA ligase suggesting that long-patch, not short-patch, BER is the likely the preferred BER pathway in *T. kodakarensis.* In short-patch BER, a single nucleotide is incorporated prior to ligation and does not require the action of Fen1 or 5’-3’ exonuclease activity. Conversely, in long-patch BER, DNA polymerase strand displacement synthesis incorporates multiple nucleotides and is dependent upon either Fen1 or 5’-3’ exonuclease activity to remove the displaced downstream DNA flap. *T. kodakarensis* cells lacking Pol B exhibit a significant growth defect when exposed to DNA damaging agents suggesting Pol B is the DNA polymerase required for BER [71–73]. Interestingly, neither Pol B, the strand-displacing DNA polymerase, or Fen1 is essential in *T. kodakarensis* [71–73] supporting the presence of redundant pathways to complete repair.

**Fig. 8.**
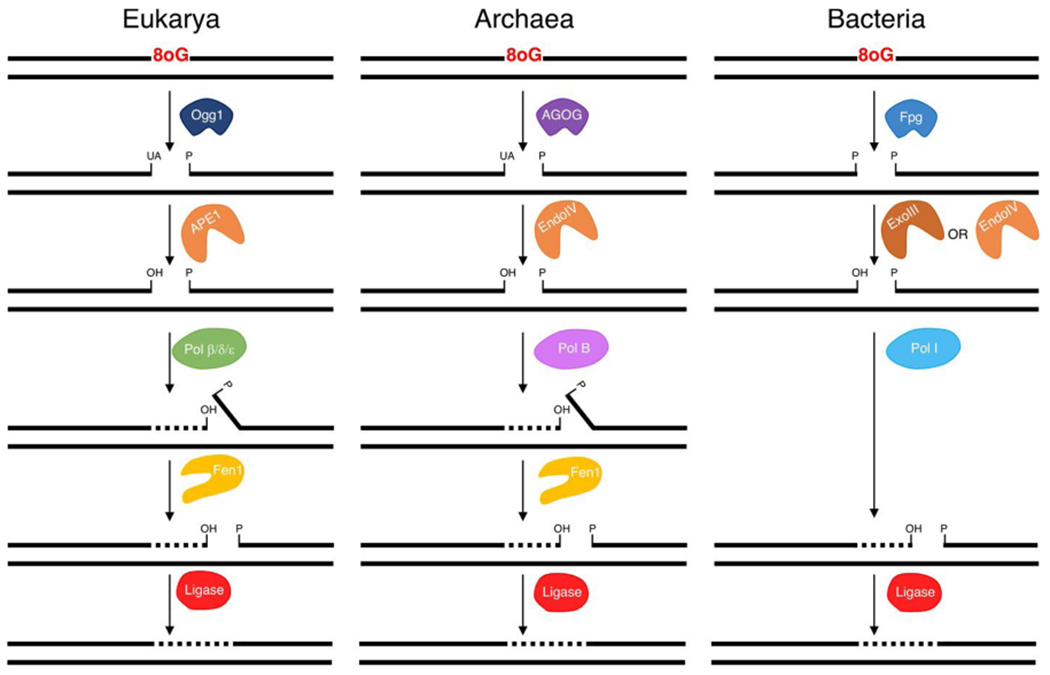
Models of 8oxoG long-patch BER in each Domain of life. 8oxo BER is initiated by a DNA glycosylase (Ogg1, AGOG or Fpg). In Eukarya and Archaea, downstream repair is carried out by an AP endonuclease (APE1 or EndoIV), DNA polymerase, Fen1 and DNA ligase. In Bacteria, downstream repair requires ExoIII or EndoIV, Pol I, and DNA ligase.

The reconstitution of archaeal BER demonstrates the overall mechanism for oxidative damage BER is conserved across all Domains (Fig. 8). In each Domain, repair is initiated by recognition of 8oxoG by a DNA glycosylase cleaving the DNA backbone and leaving either a 3’-UA (Archaea and Eukarya) or a 3’-phosphate (Bacteria) [10,12,20]. An AP endonuclease converts the 3’ termini to a hydroxyl which is then extended by a DNA polymerase. In Eukarya and Archaea long-patch BER, synthesis by DNA polymerase is strand-displacing and requires cleavage of downstream DNA by Fen1 [12,74]. While in Bacteria, Pol I functions as both the DNA polymerase and the 5’-3’ exonuclease [75]. In all Domains, the final step is nick sealing by DNA ligase.

Presently, TkoAGOG is the only 8oxoG DNA glycosylase described in *T. kodakarensis.* The deletion of TkoAGOG from the genome of *T. kodakarensis* results in a significant decrease, but not the complete loss of 8oxoG glycosylase activity in cellular extract. In *E. coli* and *S. cerevisiae*, deletion strains lacking oxidative repair enzymes are viable yet accumulate 8oxoG:A mismatches that result in G to T and C to A transversions [54–57]. Therefore, if AGOG is the only 8oxoG repair enzyme in *T. kodakarensis*, G to T and C to A transversion mutations should occur more frequently in the ΔAGOG strain. However, the mutational spectra of the parent and ΔAGOG strains are similar (Table 2) suggesting in the absence of AGOG redundant DNA repair pathways maintain genome integrity.

Redundant oxidative damage repair pathways are present in both Bacteria and Eukarya. For example, an additional DNA glycosylase, MutY, cleaves adenine in 8oxoG:A mismatch formed during replication to allow accurate repair [12,17,76,77]. In Archaea, no homologs of bacterial MutY, nor other enzymes removing adenine from a 8oxoG:A mismatch have been identified. If Archaea truly lack a dedicated enzyme to repair 8oxoG:A mismatches, it is likely the repair of 8oxoG occurs rapidly and prior to replication, thus preventing mispairing by DNA polymerases.

In addition to oxidative damage in dsDNA, the cellular nucleotide pool is also susceptible to oxidative damage. dGTP is more prone to oxidative damage compared to dG embedded in DNA [2]. Importantly, 8-oxo-dGTP can be incorporated by DNA polymerases during replication, further increasing genome instability. To deplete 8-oxo-dGTP in the cellular nucleotide pool, dedicated 8-oxo-dGTPases in Bacteria (MutT) and Eukarya (MTH1) specifically degrade 8-oxo-dGTP to 8-oxo-dGMP [25,77,78]. Archaea lack MutT homologs, and likely depend on BER to initiate 8oxoG repair. In Bacteria and Eukarya, these redundant pathways, BER, mismatch repair, and specific GTPases, target 8oxoG in the DNA or in the nucleotide pool and efficiently protect the genome against oxidative damage. Future work aims to uncover novel and redundant 8oxoG DNA repair enzymes in Archaea.

## Supplementary Material

Published Supplemental

## Figures and Tables

**Table 1:** TkoAGOG pre-steady-state rates.

Substrate	k_pss_ (min^−1^)^a^
ds8oxoG:C	1.21 ± 0.18
ds8oxoG:T	1.09 ± 0.11
ds8oxoG:A	0.55 ± 0.19
ds8oxoG:G	0.96 ± 0.08
ss8oxoG	0.41 ± 0.04

a All reactions were minimally preformed in triplicate and ± denotes standard deviation.

**Table 2:** Mutational spectrum for the ΔAGOG and TS559 strains of *T. kodakarensis*.

Strain	Total	Substitutions	A→G,T→C	G→A,C→T	A→T,T→A	A→C,T→G	G→C,C→G	G→T,C→A
ΔAGOG	2,367,080	39	44 %	28 %	0 %	5 %	15 %	8 %
TS559	5,072,211	35	14 %	31 %	0 %	9 %	26 %	20 %

## Abbreviations:

BER: base excision repair
8oG: 8-oxoguanine
Fpg: formamidopyrimidine DNA glycosylase
Ogg: 8-oxoguanine DNA glycosylase
AGOG: archaeal GO glycosylase
AP: apurinic/apyrimidinic
3’-UA: 3’-α,β-unsaturated aldehyde
3’-OH: 3’-hydroxyl
HhH: helix-hairpin-helix
5’-dRP: 5’-deoxyribose-phosphate
Pol B: DNA polymerase B
Pol D: DNA polymerase D
