# Supplementary material for: Biochemical reconstitution and genetic characterization of the major oxidative damage base excision DNA repair pathway in Thermococcus kodakarensis
Source: DNA Repair (Amst). Author manuscript; Available in PMC 2021 Apr 22. (PMC8061334; doi:10.1016/j.dnarep.2019.102767)
Supplement: Published Supplemental [file NIHMS1686612-supplement-Published_Supplemental.pdf]

Biochemical reconstitution and genetic characterization of the major oxidative damage base excision DNA repair pathway in *Thermococcus kodakarensis*.

Alexandra M. Gehring[1], Kelly M. Zatopek[1], Brett W. Burkhart[2], Vladimir Potapov[1], Thomas J. Santangelo[2], Andrew F. Gardner[1*]

[1]New England Biolabs, Inc., Ipswich, MA 01938 [2]Department of Biochemistry and Molecular Biology, Colorado State University, Fort Collins, CO 80523

SUPPLEMENTARY DATA

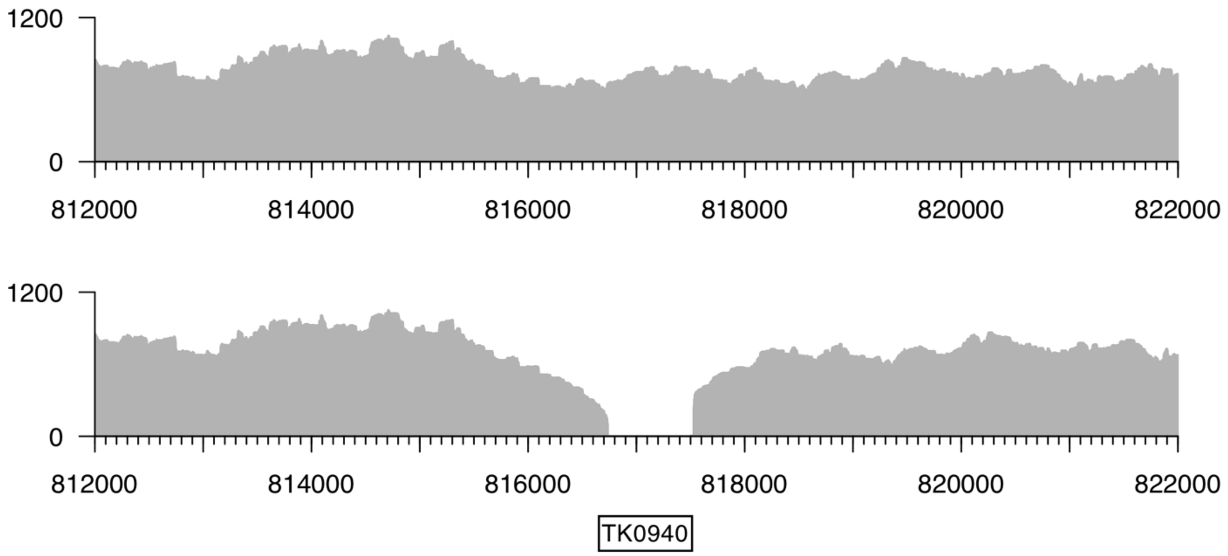

Figure S1: PacBio sequencing confirms the deletion of TK0940 which encodes AGOG. PacBio sequencing reads for libraries generated from *T. kodakarensis* ΔAGOG mapped to the reference genome using SMRT Portal. The reference sequences used were ΔAGOG (top) or TS559 (bottom). The x-axis indicates genomic position and the y-axis indicates the fold coverage at each position.

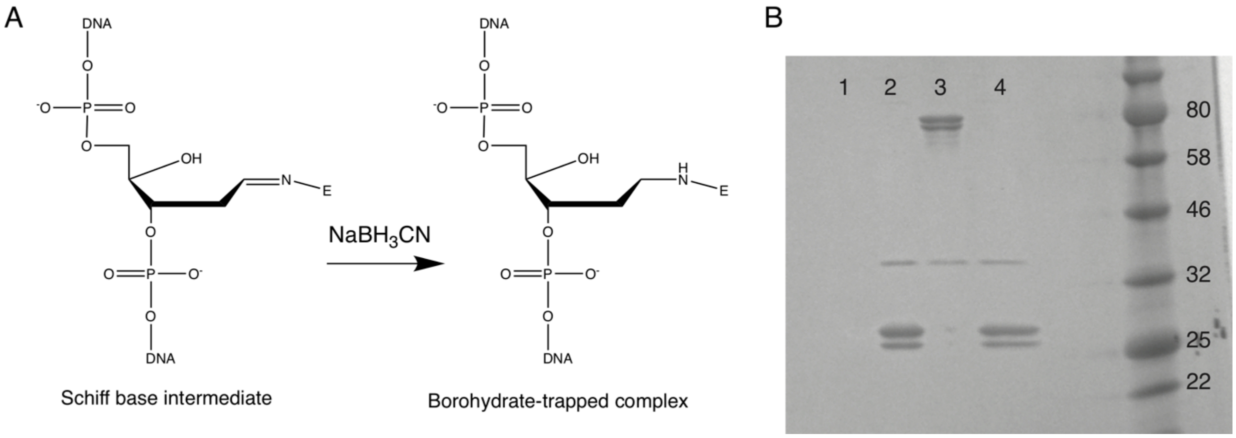

Figure S2: Trapping of TkoAGOG Schiff Base intermediate. *A,* Formation of the borohydrate-trapped TkoAGOG and DNA complex. *B,* Coomassie stained SDS PAGE showing formation of a Schiff base intermediate. Lane 1: 8oxoG DNA and sodium cyanoborohydride, Lane 2: 8oxoG DNA and TkoAGOG Lane 3: 8oxoG DNA, TkoAGOG, and sodium cyanoborohydride, Lane 4: TkoAGOG and sodium cyanoborohydride. Molecular weight standards in kDa are identified in the rightmost lane.

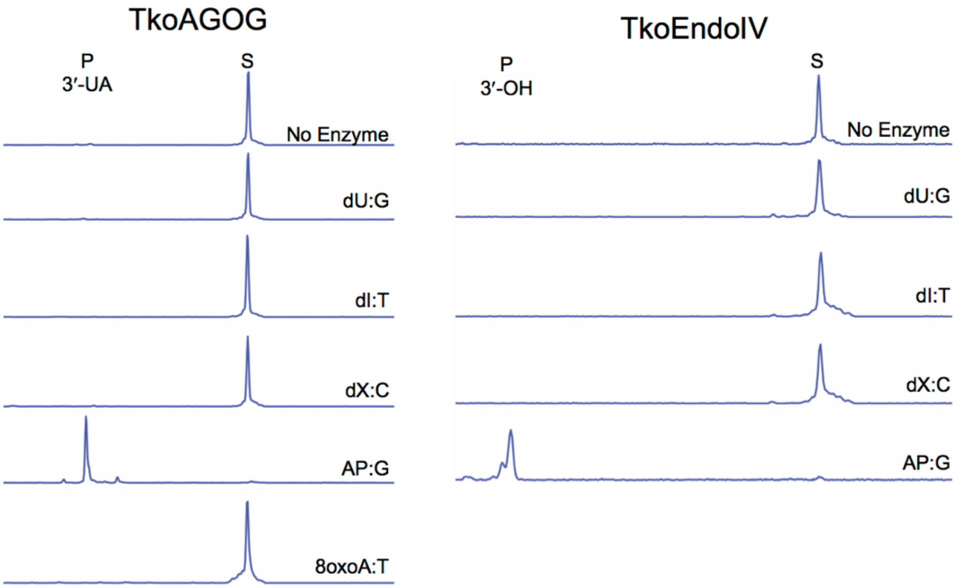

Figure S3: TkoAGOG and TkoEndoIV activity on various DNA Lesion. TkoAGOG has no activity on dsDNA containing dU, dI, dX or 8oxoA. TkoEndoIV has no activity on dsDNA containing dU, dI or dX. Both TkoAGOG and TkoEndoIV have activity on AP site containing DNA. A visualization of 60-bp dsDNA substrates containing a centralized DNA lesion incubated with either TkoAGOG or TkoEndoIV for 1 hour and then analyzed with capillary electrophoresis.

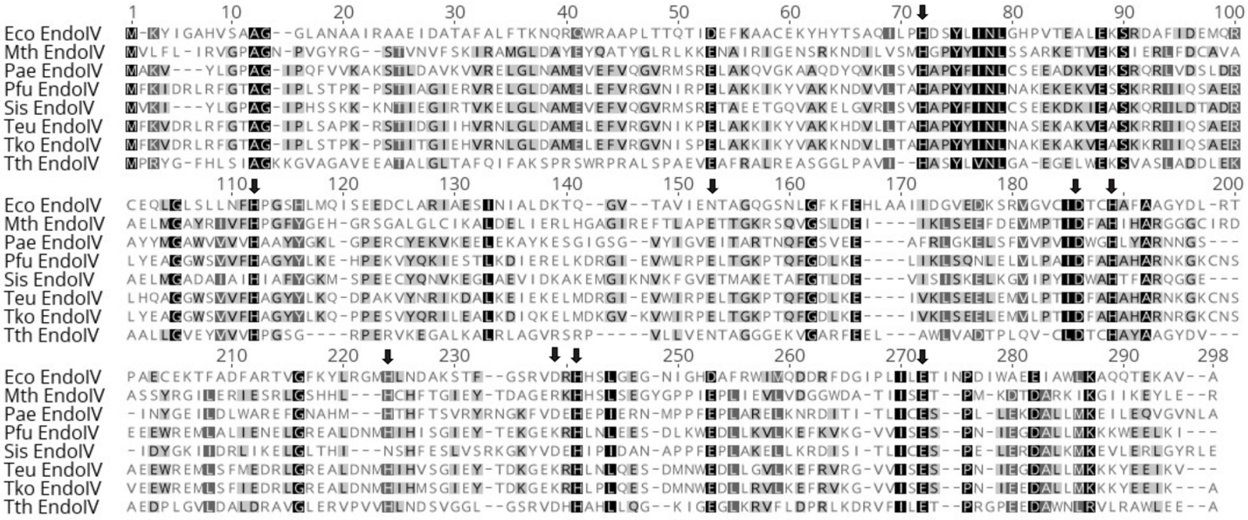

Figure S4: Amino acid sequence alignment of characterized EndoIV enzymes. The nine metal-binding active site residues are indicated by arrows. The species abbreviation and GenBank ascension numbers used are as follows: Bacteria: Eco, *Escherichia coli* (NP_416664.1); Tth, *Thermus thermophilus* (AAS80830.1); Archaea: Tko, *Thermococcus kodakarensis* (BAD84359); Teu, *Thermococcus eurythermalis* (WP_050002723.1); Pfu, *Pyrococcus furiosus* (AAL80382.1); Mth, *Methanothermobacter thermautotrophicus* (AAB85506.1); Pae, *Pyrobaculum aerophilum* (AAL64792.1); Sis, *Sulfolobus islandicus* (ADX86710.1).

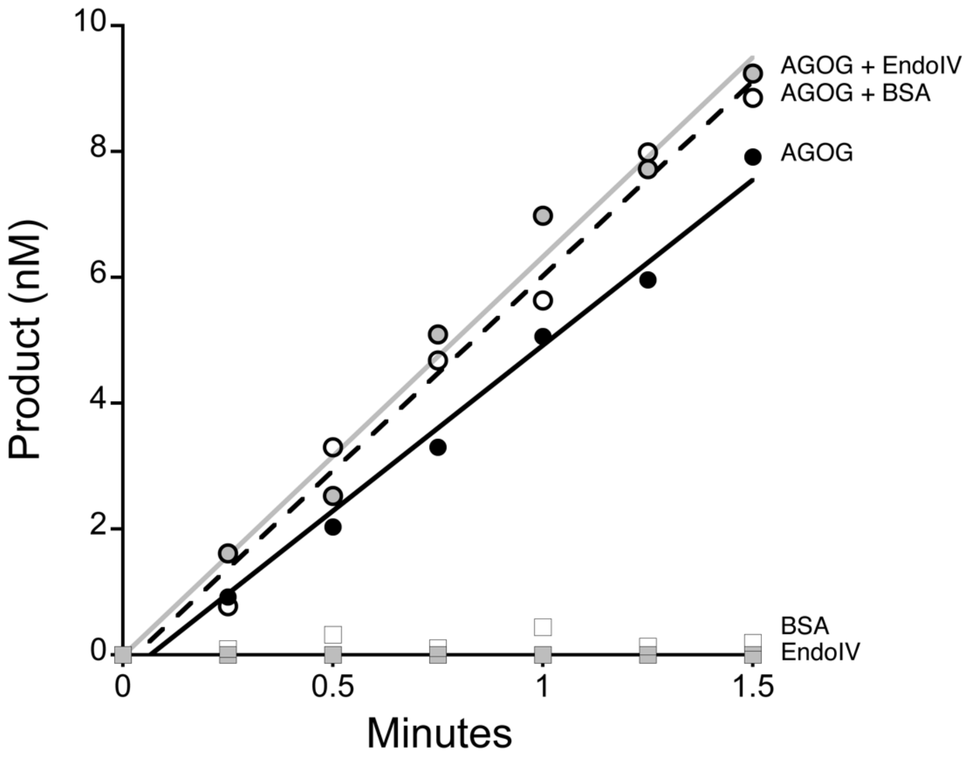

Figure S5. TkoEndoIV does not stimulate TkoAGOG activity. An 8oxoG-containing substrate was incubated with AGOG (5 nM) alone (black filled circles), with BSA (5 nM) (open circles) or TkoEndoIV (10 nM) (grey filled circles) at 65°C and quenched with formamide and EDTA at various times (as described in the Material and Methods section). The rate of AGOG cleavage (5.3 min^{-1}) was not significantly effected by addition of TkoEndoIV (6.3 min^{-1}) or BSA (6.2 min^{-1}). As controls, TkoEndoIV (10 nM) (open squares) or BSA (5 nM) were incubated with 8oxoG-containing substrate and showed no activity.

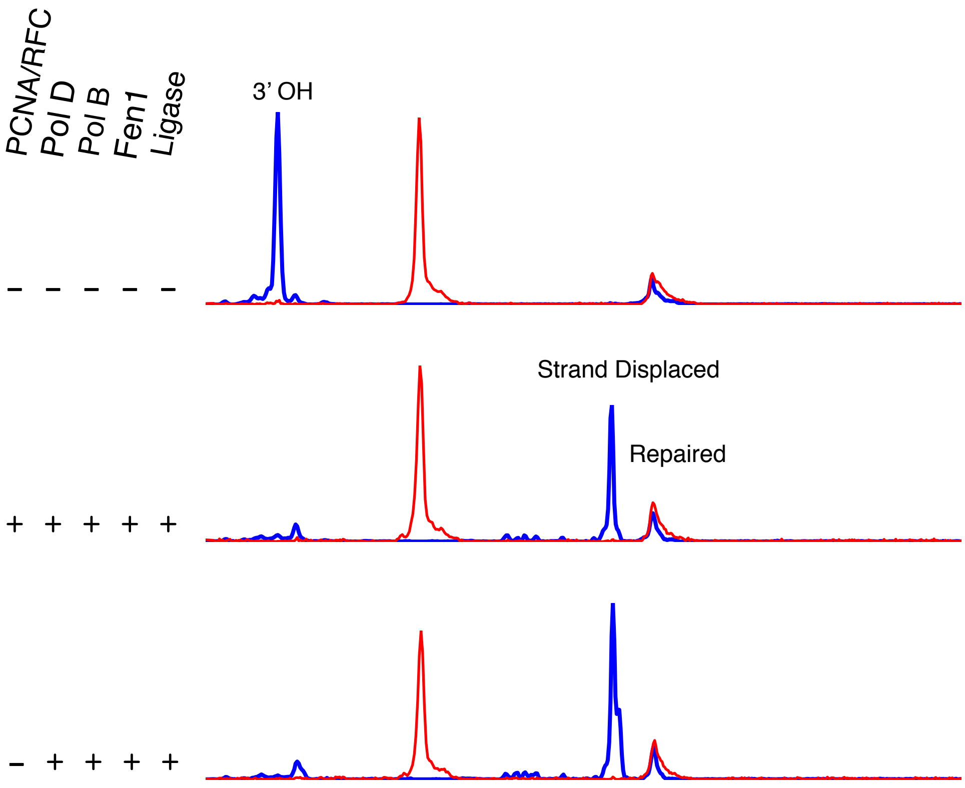

Figure S6: DNA substrates were pre-incubated with TkoAGOG and TkoEndoIV leaving a 3′-OH with a FAM (blue) label. DNA replication proteins were then added as shown on the left and incubated at 65°C for 15 minutes. The repair was monitored by the appearance of the 60-nt dual-labeled FAM/ROX product (repaired) and the 60-nt FAM only labeled product (strand displaced).
